# Polarised Asymmetric Inheritance of Accumulated Protein Damage in Higher Eukaryotes

**DOI:** 10.1371/journal.pbio.0040417

**Published:** 2006-12-05

**Authors:** María A Rujano, Floris Bosveld, Florian A Salomons, Freark Dijk, Maria A.W.H van Waarde, Johannes J.L van der Want, Rob A.I de Vos, Ewout R Brunt, Ody C.M Sibon, Harm H Kampinga

**Affiliations:** 1 Department of Cell Biology, Section of Radiation and Stress Cell Biology, University Medical Center Groningen, University of Groningen, Groningen, The Netherlands; 2 Department of Cell Biology, Section of Electron Microscopy, University Medical Center Groningen, University of Groningen, Groningen, The Netherlands; 3 Pathology Laboratory Oost Nederland, Enschede, The Netherlands; 4 Department of Neurology, University Medical Center Groningen, University of Groningen, Groningen, The Netherlands; University of California San Francisco, United States of America

## Abstract

Disease-associated misfolded proteins or proteins damaged due to cellular stress are generally disposed via the cellular protein quality-control system. However, under saturating conditions, misfolded proteins will aggregate. In higher eukaryotes, these aggregates can be transported to accumulate in aggresomes at the microtubule organizing center. The fate of cells that contain aggresomes is currently unknown. Here we report that cells that have formed aggresomes can undergo normal mitosis. As a result, the aggregated proteins are asymmetrically distributed to one of the daughter cells, leaving the other daughter free of accumulated protein damage. Using both epithelial crypts of the small intestine of patients with a protein folding disease and *Drosophila melanogaster* neural precursor cells as models, we found that the inheritance of protein aggregates during mitosis occurs with a fixed polarity indicative of a mechanism to preserve the long-lived progeny.

## Abstract 2

Human cells containing polyglutamine damage enter mitosis and complete cytokinesis. The association of aggresomes with one centrosome means that accumulated damage is asymmetrically inherited in only one daughter cell.

## Introduction

Disease-associated, mutant misfolded proteins such as cystic fibrosis transmembrane conductance regulator [1], peripheral myelin protein 22 [2], huntingtin (Htt) [3], parkin [4], α-synuclein [5,6], ataxin-3, and Down syndrome critical region 1 [7] are found to accumulate in aggresomes. Aggresomes are defined as pericentriolar cytoplasmic inclusions that contain misfolded ubiquitinated proteins ensheathed in a cage of intermediate filaments. Aggresomes are formed by an ordered process that involves retrograde transport of preformed microaggregates from the periphery of the cell specifically to the centrosomes, or to the microtubule organizing center (MTOC) in higher eukaryotes [1,8]. Aggresomes arise when the capacity of cells to degrade misfolded proteins by the ubiquitin–proteasome system is exceeded [9]. Once sorted to the aggresome, protein aggregates can no longer be degraded by proteasomes [10]. Instead, some cells can dispose of aggresomes by autophagy [11–13], thereby avoiding their enlargement and consequent saturation of the system to an extent that becomes noxious for the cell. By using this temporal storage-disposal mechanism, cells can maintain their function and integrity for prolonged periods of time [14]. Likewise, during aging, cells in all biological systems accumulate oxidised proteins, e.g., carbonylated proteins, which form high–molecular weight aggregates that escape degradation [15]. Cells like self-renewable tissue stem cells or germ-line cells that exist in the tissue during the entire lifespan may use storage of aberrant proteins in aggresomes to preserve fitness and functionality. Because aggresomes are formed at the MTOC, which is an organelle crucial for proper spindle formation and cell division, it is conceivable that aggresome formation and irreversibly accumulated proteins interfere with proper cell division and hence impair cell renewal in proliferating tissues. However, we now show that the accumulation of misfolded proteins in aggresomes still allows normal cell divisions in which irreversibly damaged, stored proteins are inherited by only one daughter cell. This asymmetric inheritance appears to be evolutionarily conserved and occurs with a stringent polarity in progenitor cells, preventing transmission of damaged proteins to the longest lived daughter cell.

## Results

**Figure 1:**
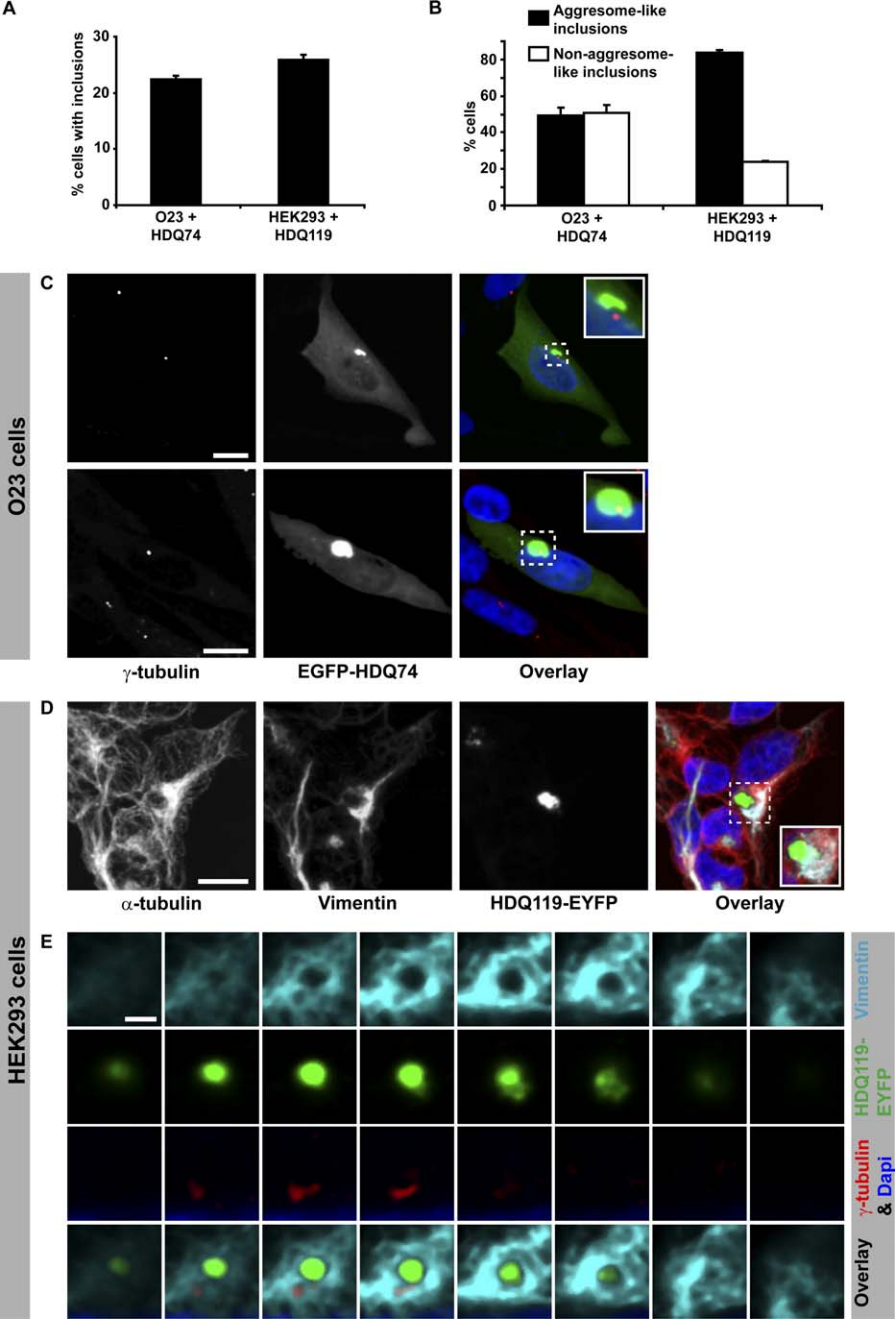
Polyglutamine-Expanded Proteins Form Aggresomes in Hamster O23 Cells and Human HEK293 Cells (A) Percentage of cells containing inclusions 24 h after transfection with a fluorescently tagged huntingtin fragment containing a stretch of either 74 (O23: EGFP-HDQ74) or 119 (HEK293: HDQ119-EYFP) glutamines. (B) Fraction of cells showing either aggresome-like inclusions or non–aggresome-like inclusions (nuclear and/or multiple scattered inclusions). Bars represent standard errors of the mean. (C) Aggresome-like inclusions are either close to (upper panel) or co-localise (lower panel) with the centrosomes (decorated with γ-tubulin antibodies) in interphase O23 cells (likewise in HEK293 cells, Figure S1). (D) Vimentin microfilaments are redistributed in a cage-like manner around the inclusion, consistent with aggresome morphology. Note that also microtubules (decorated with α-tubulin antibodies) showed partial redistribution to the aggresome. (E) Sequential confocal planes of an aggresome showing both co-localisation with the centrosome and the cage of vimentin. For a full image of this cell see Figure S1B. DNA is stained with DAPI (blue) and only shown in the overlay images. Bars in (C) and (D) represent 10 μm. Bar in (E) represents 2 μm.

To induce the formation of intracellular inclusions in mammalian cells, we transiently expressed a fluorescently-tagged fragment of the exon-1 of the *huntingtin (Htt)* gene, either containing 74 (HDQ74) or 119 (HDQ119) glutamine repeats, in hamster O23 cells and in human embryonic kidney 293 (HEK293) cells. Whereas the expression of HDQ74 resulted in up to 22% of O23 cells with inclusions after 24 h of expression (Figure 1A), in HEK293 cells, inclusion formation was nearly absent with this polyglutamine stretch (unpublished data), indicating that the protein quality control of HEK293 cells is more efficient than that of O23 cells. However, when transfected with the longer polyglutamine stretch (HDQ119), HEK293 cells showed a similar amount of cells with inclusions (26%) after 24 h of expression (Figure 1A). In all further experiments, we therefore used HDQ74 for O23 and HDQ119 for HEK293 cells.

Distinct types of inclusions were observed in both cells lines: aggresome-like perinuclear inclusions and non–aggresome-like inclusions. The latter includes cells that contain multiple cytoplasmic and/or nuclear inclusions. In O23 cells, the fraction of cells with aggresome-like perinuclear inclusions was almost 50%, whereas in HEK293, more than 80% had formed an aggresome-like perinuclear inclusion (Figure 1B). These results suggest that HEK293 cells not only are less prone to inclusion formation than O23 cells are, but also seem more efficient in transporting unfolded polyglutamine proteins to the MTOC to form aggresomes.

To determine whether the perinuclear inclusions can be indeed classified as aggresomes, transfected cells were fixed and immunolabelled for centrosomal and cytoskeletal proteins. Confocal laser scanning microscopy revealed colocalisation or close proximity of the inclusions to the centrosomal marker γ-tubulin (average distance <2 μm) in a fraction of both O23 (Figure 1C) and HEK293 cells (Figure 1E and Figure S1A). Furthermore, partial reorganisation of vimentin microfilaments into a cage-like structure surrounding the inclusion was observed (Figures 1D and 1E and Figure S1B). These data show that the perinuclear inclusions formed in O23 and HEK293 cells are aggresomes with similar characteristics to those formed in a dynein-dependent manner, e.g., after the expression of misfolded mutant cystic fibrosis transmembrane conductance regulator [1,8]. In contrast, the nuclear inclusions and/or scattered secondary inclusions seen in the remaining fraction of cells (50% in O23, 20% in HEK293) were not associated with γ-tubulin (Figure S2) and did not show reorganisation of vimentin (unpublished data). These cells also often contained fragmented nuclei that were not seen in aggresome-containing cells (unpublished data). Because HEK293 cells, which are more efficient in forming aggresomes (Figure 1B), are also less prone to aggregate formation by polyglutamine proteins (Figure 1A, unpublished data) we suggest that the nonaggresomal inclusion-containing cells may represent cells with a more severe phenotype in which the process of aggresome formation and clearance was already saturated.

**Figure 2:**
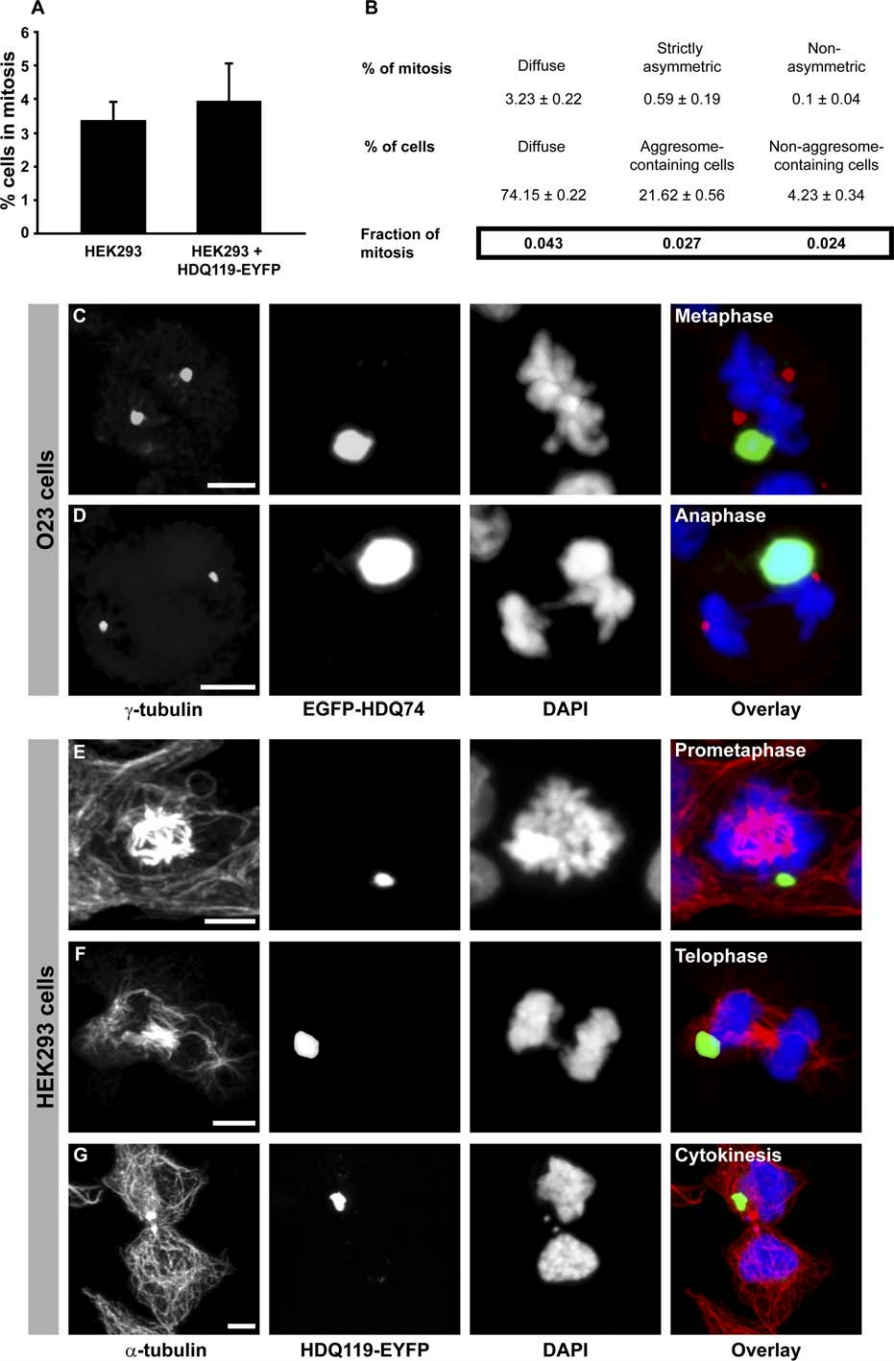
Aggresomes Do Not Impair Mitotic Cell Division (A) Quantitative analysis of total mitoses in wild-type HEK293 cells and polyglutamine-expressing HEK293-HDQ119 cells. Bars represent standard error of the mean. (B) Relative fraction of mitoses in each population (diffuse, aggresome-containing, and non–aggresome-containing) of HEK293-HDQ119 cells. (C–G) Representative pictures of fixed O23 (C and D) and HEK293 (E–G) aggresome-containing cells in different mitotic phases. The aggresome is associated with only one of the poles during metaphase, anaphase, and telophase. (C) shows that alignment of chromosomes in metaphase appears normal, and (D and F) show that segregation during anaphase-telophase appears to be normal. Similarly, (C and D) show that positioning of the centrosomes is normal, and (E–F) show that distribution of microtubules and (G) cytokinesis are normal. DNA is stained with DAPI (blue). Bars, 5 μm.

We then analysed the ability of inclusion-containing cells to enter into and progress through mitosis. The overall percentage of mitotic HEK293 cells was similar between nontransfected and HDQ119-expressing HEK293 cells (Figure 2A). Because only part of the transfected cells contain aggresomes (Figure 1), we next determined the fraction of mitotic transfected cells that had the following characteristics: (i) they had not yet formed inclusions (diffuse), (ii) they had inclusions that were strictly asymmetrically distributed, or (iii) they had multiple, nonasymmetrically distributed inclusions. As can be seen in Figure 2B, the fraction of mitotic cells was equal irrespective of the type of inclusions that had formed. This finding indicates that the different inclusions did not induce a cell-cycle checkpoint to prevent entry into mitosis. However, in these fixed samples, mostly the early phases of mitosis (prometaphases and metaphases) (unpublished data) could be detected in the population of cells with multiple nonasymmetric inclusions, suggesting that they are unable to normally progress through mitosis. This was further supported by detailed examination of α-tubulin in one of a few rare mitotic cells with multiple inclusions that did progress to anaphase. In such a cell, the spindle midbody, which is formed before the formation of the contractile ring, which is necessary to complete cytokinesis, was substantially thickened and disorganised (Figure S2B). In contrast, in both O23 (Figure 2C and 2D) and HEK293 (Figure 2E–2G), aggresome-containing mitotic cells were found with normal appearance in all phases of mitosis, including prometaphase (Figure 2E), metaphase (Figure 2C), anaphase (Figure 2D), and telophase (Figure 2F). The aggresomes in all of these cells were clearly associated with one of the spindle poles and were localised in close proximity to the MTOC (average distance <2 μm); therefore, these mitoses were asymmetric. Remarkably, centrosome positioning (Figure 2C and 2D) and microtubule arrangement (Figure 2E–2G) were not affected by aggresomes. Also, aggresome-containing cells were detected that had nearly completed mitosis (Figure 2G) and that showed normal midbody formation (Figure S2B), suggesting that these cells not only enter but also normally exit mitosis.

**Figure 3:**
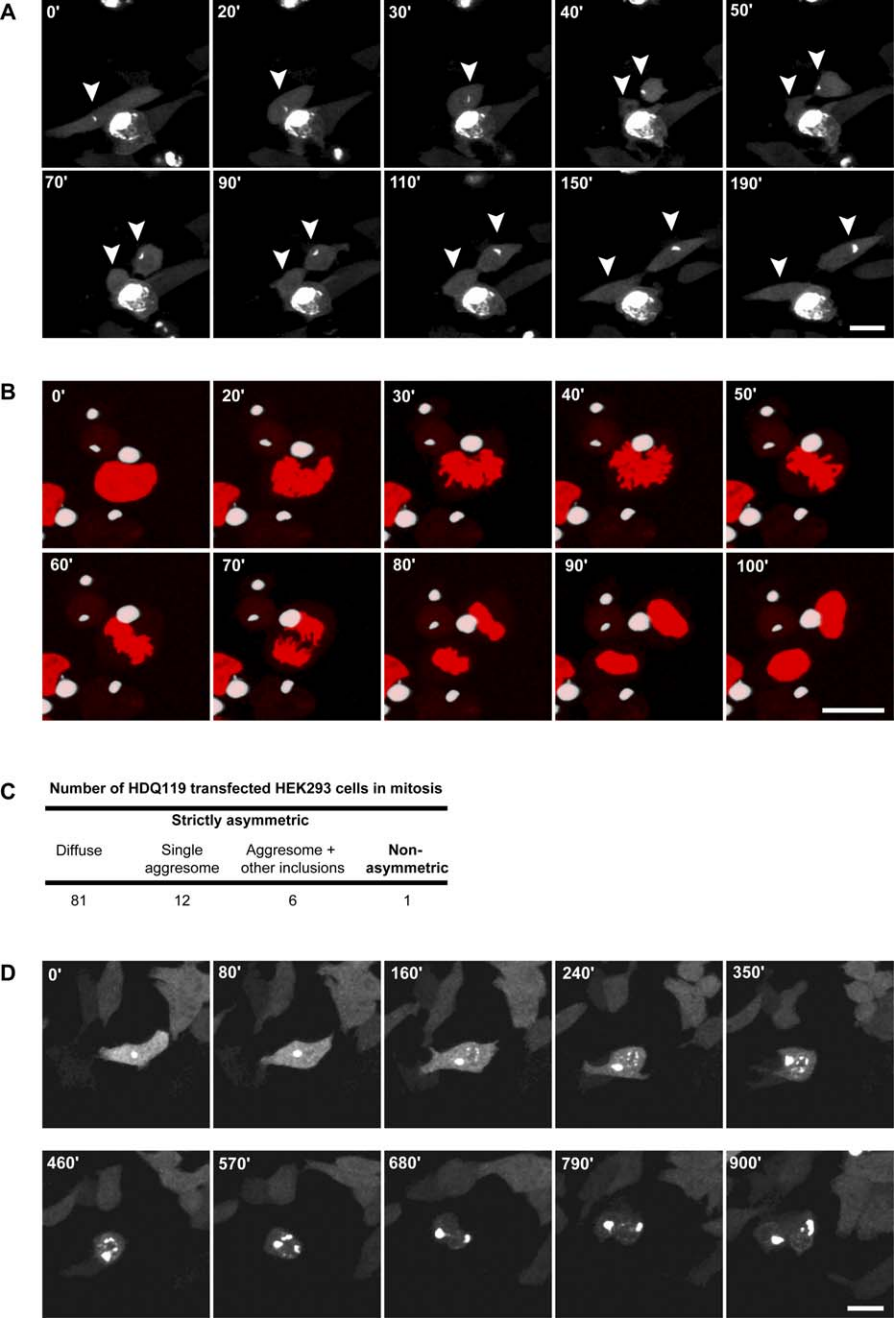
Aggresome Formation Does Not Impair Cell Division and Leads to Asymmetric Inheritance of Aggregated Proteins (A) Time-lapse imaging of O23 mitotic cells expressing EGFP-HDQ74. An aggresome (indicated by the arrowhead) containing cell enters mitosis (frame 30') and completes division in 40 min (frame 70') without any apparent irregularities. The two resulting daughter cells are marked by arrowheads. See also Videos S1 and S2. (B) Time-lapse imaging of HEK293 mitotic cells expressing HDQ119-EYFP. The aggresome containing cell enters mitosis (frame 20') and like in O23 cells, completes cytokinesis (frame 90') without irregularities. Note that mitosis in HEK293 cells is slower than in O23 cells (60 ± 10 min); although the timing is normal compared to non-transfected HEK293 or cells with diffuse polyglutamine protein. See also Videos S3 and S4). (C) Distribution of mitoses in a total of 100 HDQ119 transfected HEK293 cells recorded. (D) A cell with multiple inclusions enters mitosis (frame 350') and completes cytokinesis with a delay of approximately 510 min (frame 900'). Both daughter cells inherit multiple inclusions and do not show normal morphology (see also Video S5). All images are selected frames of the time-lapse recording shown in Videos S1, S2, and S5. Videos of dividing cells without inclusions are shown in S6 (O23) and S7 (HEK293). Bar, 20 μm.

To determine conclusively whether mitotic cells containing aggresomes are able to complete cytokinesis without abnormalities, we performed time-lapse imaging on living cells. These data showed that cells with aggresomes indeed not only were capable of entering mitosis but also were able to complete cytokinesis without any irregularities or delay. The time to complete mitosis was 45 (±5) min for O23 cells with aggresomes compared to 40 (±9) min for cells without any inclusions. In HEK293 cells, mitosis lasted 70 (±10) min for aggresome-containing cells compared to 65 (±10) min for non–inclusion-containing HEK293 cells (Figures 3A–3C and Videos S1–S4, S6, and S7). In cells with a more severe phenotype, i.e., with scattered multiple inclusions, the completion of mitosis was strongly delayed (550 ± 50 min; Figure 3D and Video S5) or even failed completely (unpublished data).

In aggresome-containing cells, only one of the daughter cells inherited the accumulated protein damage. Because this daughter cell continued to produce more misfolded, transgene-encoded, polyglutamine-expanded protein, it often progressed into the more severe phenotype with secondary inclusions. Such cells subsequently lost their morphology and detached (unpublished data). The inclusion-free daughter cells persisted in culture with normal morphology during the time of recording, and in many cases, de novo aggresome formation was observed due to the continuation of expression of the mutant protein. Together, these results demonstrate that cells with aggregated polyglutamine-expanded proteins are capable of successfully completing mitosis after sorting misfolded aggregated proteins to the MTOC.

**Figure 4:**
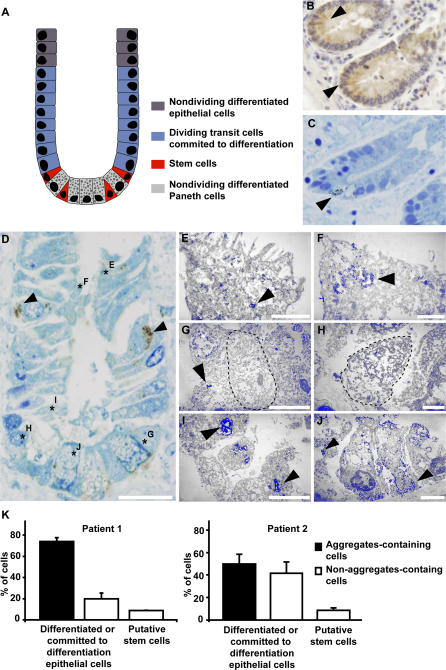
Polyglutamine Aggregates Are Present in Committed Crypt Cells but Absent in Stem Cells in the Small Intestine of SCA3 Patients (A) Schematic representation of an intestinal crypt for visualisation of the different cell types present in this tissue. Light micrographs of a 4-μm (B) and 1-μm (C) section of a crypt from an SCA3 patient showing positive staining for anti-Musashi antibody. Note that stem cells are localised in between and on both sides of the morphologically recognizable Paneth cells residing at the base of the crypt. (D) A light micrograph of a crypt of a SCA3 patient showing positive staining for the anti-polyglutamine antibody IC2 in some epithelial cells (arrowheads). The asterisks (marked E–J) show representative positions of cells analyzed by subsequent electron microscopy. (E–J) show digitally modified, pseudo-coloured images of electron micrographs indicating the polyglutamine staining in blue. Differentiated epithelial cells (E), transit epithelial cells (F and I), and Paneth cells (J) contain polyglutamine aggregates (arrowheads). Stem cells (G) are negative for polyglutamine aggregates but occasionally contain micro-aggregates (H). Note that also some electron dense material is stained blue by this digital processing. In (G and H), contours are provided in black dashed lines to indicate the stem cells. Bars: D, 20 μm; E and H, 2 μm; F, 1 μm; G, I and J, 5 μm. (K) Quantification of cells with aggregates in the crypts of two SCA3 patients. As double labelling for aggregates and stem cells failed, only the stem cells that were adjacent to the Paneth cells were counted, because these could be easily identified on this basis.

Aggresomes remain linked to only one centrosome during cell division, which raised the question whether asymmetric inheritance of protein aggregates also occurs in dividing cells of polarised tissues and what the implications could be of such an asymmetry. Asymmetric cell division is intrinsically linked to progenitor cells and is a conserved mechanism responsible for the generation of different cells fates in both unicellular and multicellular organisms, especially during development [16,17]. This differentiation is achieved by asymmetric localisation of cell fate determinants in the progenitor cells during mitosis [18]. To investigate whether and how the inheritance of aggregated proteins is organised in polarised tissue, we analysed crypts of the small intestine from two patients with the human neurodegenerative disease spinocerebellar ataxia type 3 (SCA3, Machado-Joseph disease, Figure 4). Although intestinal dysfunction is not obvious in patients with SCA3, this tissue provides an elegant model to investigate the tissue distribution of accumulated mutant ataxin-3 polyglutamine protein during the individual's lifespan. Intestinal tissue contains stem cells that give rise to short-lived committed progenitors and differentiated cells. The SCA3 intestinal mucosal crypts were immunostained for the presence of polyglutamine aggregates with polyclonal anti–ataxin-3 or monoclonal anti-polyglutamine IC2 antibodies. Using both light and electron microscopy, we found that in both patients, committed differentiated epithelial cells (Figure 4D and 4E), dividing transit cells (Figure 4F and 4I), as well as the adjacent nondividing differentiated Paneth cells (Figure 4J) in the crypts contained large polyglutamine cytoplasmic inclusions as recognised by the anti-polyglutamine antibody. Similar data were obtained with the anti–ataxin-3 antibody (unpublished data). These inclusions were not detected in the intestinal crypts of healthy controls, and moreover, the expression levels of the ataxin-3 protein seemed equal in all cell types, including the putative stem cells (see below).

Stem cells lie at the base of the crypts and are thought to be localised adjacent to or in between the Paneth cells [19,20] (Figure 4A). We explored the latter by analysing the crypts for the presence of Musashi-1, an RNA-binding protein that is important for the maintenance of neural stem cells. In agreement with earlier data [21–23], the Musashi-1 antibody indeed decorated cells adjacent to or in between the Paneth cells (Figure 4B and 4C), further supporting the idea that these are the stem cells within the intestinal epithelium. Because double staining with the Musashi-1 antibody and the anti-polyglutamine or anti–ataxin-3 antibody was not possible, we identified putative stem cells in the crypts on the basis of their localisation next to the Paneth cells. In these putative stem cells, no polyglutamine inclusions were detected (Figure 4G and 4H). In fact, the percentage of non–stem cells with aggregates in the crypts of both patients was strikingly high, whereas stem cells containing aggregates were never detected (Figure 4K). This indicates that in both patients (aged 87 y and 64 y at time of death), intestinal stem cells that were supposed to have accumulated mutant ataxin-3 over the course of patients' entire lives were devoid of large aggregates, whereas the short-lived committed and differentiated cells did contain such polyglutamine aggregates. Similar results were found in skin tissues (unpublished data). Ultrastructural analysis further supported the light microscopy data that the absence of inclusions in the stem cells was not due to lack of expression of the polyglutamine protein. In fact, in the electron microscope analysis, occasional micro-aggregates were detected in these stem cells (Figure 4H). Together, these patient data are consistent with a model in which aggregated proteins are asymmetrically distributed during mitosis and inherited by the non–stem cell after division. However, as mitotic stem cells were not detected in this patient material, this hypothesis could not be verified.

**Figure 5:**
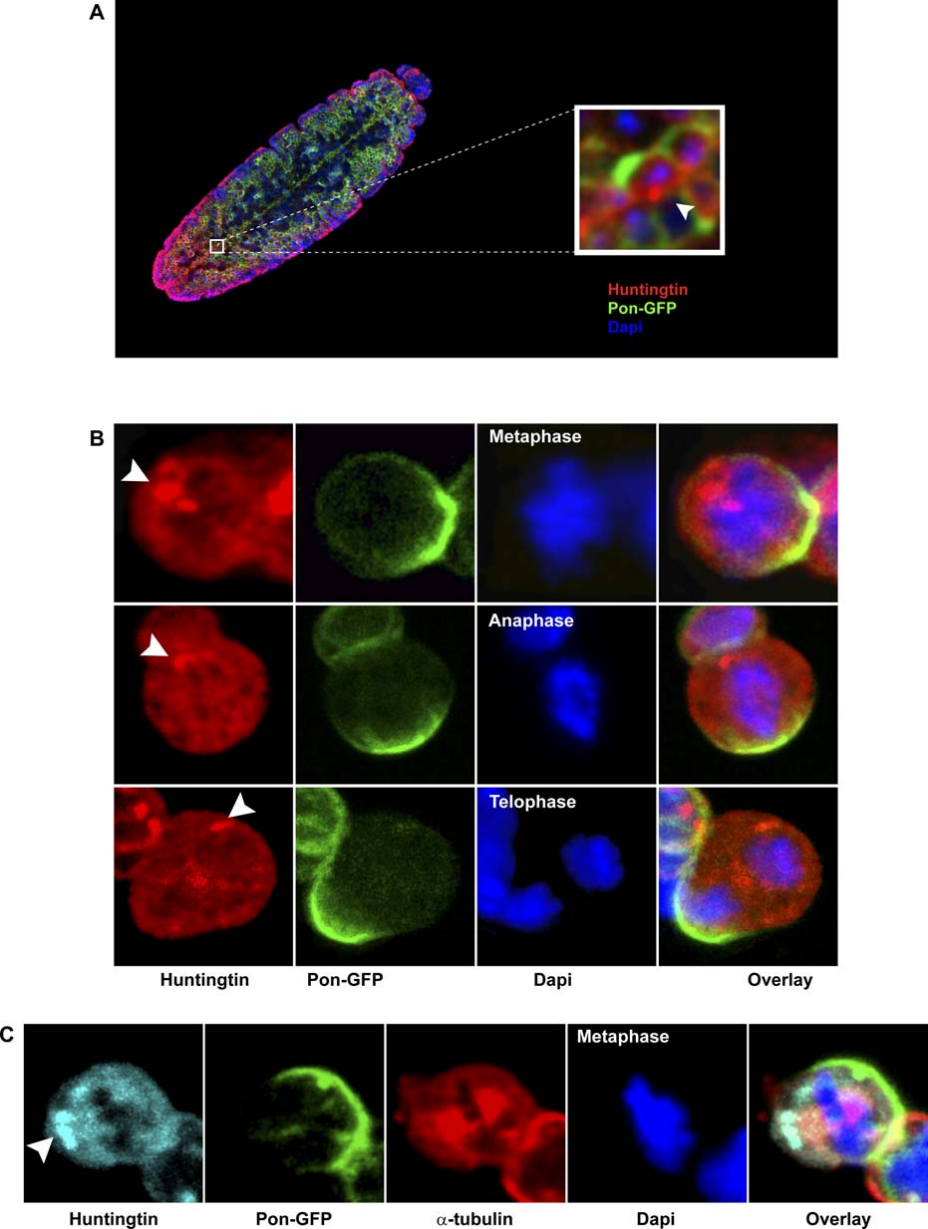
Polyglutamine Aggregates Are Inherited by De Novo Generated Neuroblast Cells after Mitosis in *D. Melanogaster* (A) Expression of Htt-Q128 (red) and Pon-GFP (green) was assessed by confocal laser scanning microscopy in whole embryos (Stage 11, in which anterior is at the top). Occasionally, Htt-Q128 aggregates were observed (inset). (B) During mitosis, the aggregated protein Htt-Q128 is associated with only one of the poles in metaphase, anaphase, and telophase, opposing the Pon-GFP crescent, indicative of asymmetric inheritance to de novo generated neuroblast. (C) Spindle pole–associated aggregates were more clearly visualised after α-tubulin (red) staining in Htt-Q128 (cyan), Pon-GFP (green) neuroblasts. DNA is stained with DAPI (blue).

To investigate more directly whether the asymmetric inheritance of misfolded proteins occurred with specific polarity, we used D. melanogaster embryonic neuroblasts as a model. These neuroblasts are well-studied stem cells that divide asymmetrically to give rise to another neuroblast and a fate-committed smaller daughter cell called ganglion mother cell (GMC). Whereas the neuroblast undergoes a limited number of cell divisions and subsequently dies by apoptosis before the end of embryogenesis, the GMC divides only once more and generates a pair of neurons or glial cells, which will persist during the entire fly lifespan [18,24]. To investigate the distribution of aggregated proteins in progenitor cells, we generated a recombinant fly expressing the N-terminal fragment of human Htt that contains 128 glutamine repeats (Htt-Q128) [25] and the adapter protein Partner of Numb fused to green fluorescent protein (Pon-GFP) [26]. The Pon protein segregates asymmetrically during neuroblasts mitosis to the GMC [27] and was used here to identify the committed daughter cell. The Gal4-UAS binary system [28] was used to express Pon-GFP and Htt-Q128 (UAS-Pon-GFP, UAS-Htt-Q128) under the control of the *neuralized* promoter, which drives strong Gal4 expression in neuronal precursor cells [29]. Htt antibody labelling of whole embryos revealed expression of Htt-Q128 and occasional cytoplasmic aggregates in cells of the embryonic central nervous system that also expressed the Pon-GFP protein (Figure 5A). Next, isolated embryonic neuroblasts expressing both Htt-Q128 and Pon-GFP were analysed using confocal microscopy. Mitotic neuroblasts were identified by the localisation of Pon-GFP in a cortical crescent and the presence of condensed DNA, visualised by DAPI staining. Although the Htt-Q128 protein was expressed in all the Pon-GFP–positive mitotic neuroblasts, only a minor fraction showed inclusions. This small fraction is likely due to short expression time of the protein before isolation of the neuroblasts (1–5 h). The aggregated protein was usually confined within a single inclusion body, associated with one of the spindle poles, resembling the aggresome that we observed in the mammalian cell model (Figure 1A). Most importantly, in 100% of the mitoses that we could score (*n* = 12), the aggresome-like inclusion exclusively segregated to the de novo–generated neuroblast (Figure 5B), resulting in a protein damage–free GMC. Additional α-tubulin staining further substantiated the spindle pole association of the aggregates (Figure 5C). These results show that in D. melanogaster neural precursor cells, asymmetric distribution of misfolded aggregated proteins occurs with a polarity that favours the fate-committed, long-lived progeny.

## Discussion

The data presented here demonstrate that cells in higher eukaryotes that have accumulated irreversible protein damage transport it to the MTOC, where it is stored in aggresomes. These cells are able to mitotically divide, resulting in unequal inheritance of the aggresome to one daughter cell after asymmetric cell division. Our results further demonstrate that the segregation of protein aggregates occurs with a fixed polarity during development and suggest that this may also happen in somatic cell differentiation.

It was anticipated that aggresome formation would impair cell division [1]. Indeed, it was previously found that heat-induced protein damage to the MTOC strongly hindered progression through mitosis and/or resulted in mitotic errors [30,31]. However, we show for the first time, to our knowledge, that cells that have orderly formed aggresomes in response to the accumulation of misfolded proteins enter and exit mitosis without significant delay or errors. Apparently, the entire apparatus needed for mitosis is not disturbed in aggresome-containing cells, unlike in cells with a more severe phenotype with multiple scattered inclusions. We cannot completely rule out that cells with small, nonaggresomal inclusions can also normally progress through mitosis. Experiments to disrupt aggresome formation by overexpression of p50 dynamitin or microtubule-destabilizing drugs failed because they also affected mitosis without expression of polyglutamine proteins. However, small aggregates that were not closely associated to the MTOC were not detected in the fixed material nor in the life recording (late phase) mitotic cells. Hence, the storage of accumulated damage in aggresomes does not hinder mitosis, but rather enables the cells to divide asymmetrically and transmit accumulated, nonfoldable aggregated proteins to only one of the daughter cells.

The mechanism driving the asymmetric segregation of damaged proteins and its polarity remains to be elucidated. Centrosomes, polarised microtubules, and the actin cytoskeleton are critical structures in the unequal distribution and inheritance of cell fate determinants in *Caenorhabditis elegans, D. melanogaster,* and the mollusc *Ilyanassa obsoleta* [32,33]. It is likely that asymmetric segregation of damaged proteins accumulated in aggresomes is linked to the intrinsic differences between replicated centrosomes [34]. Centrosome duplication itself is asymmetrical because only one of the two daughter centrosomes inherits the pericentriolar matrix that surrounds the centrioles [35]. Lambert and Nagy [36] demonstrated that the asymmetric inheritance of mRNAs for several patterning genes was dependent on the targeting of the mRNAs to the pericentriolar matrix in a microtubule-dependent manner. Our findings that the asymmetrically inherited misfolded proteins are first transported to the MTOC, which was proven to be a dynein-dependent [8] process, strongly suggest that the asymmetric inheritance of pericentriolar matrix enables an asymmetric distribution of aggregated proteins.

The asymmetric inheritance of accumulated protein damage may have important biological implications during development, somatic tissue differentiation, and ageing. In the unicellular organism *Saccharomyces cerevisiae,* age asymmetry was suggested to be dependent on partitioning of undamaged cellular components to the progeny [37]. Indeed, aged mother cells showed markers of oxidative stress [38], and this was recently linked to asymmetric sorting of oxidatively damaged proteins to the “mother” cell during cytokinesis, thus providing the most favourable situation for the daughter cell [39]. Similarly, in *Escherichia coli,* asymmetric inheritance of cellular damage has been related to ageing: the cell that inherits the old pole grows more slowly, has decreased offspring, and has an increased incidence of death, suggesting asymmetric distribution of damage [40]. In line with this, our data from D. melanogaster neuroblasts now show that the progenitor cell, which divides only a limited number of times, inherits the aggregated proteins, thus ensuring that the sibling GMC, which divides into two long-lived cells of different fates (neurons and glia), stays free of accumulated damage. So, also in *Drosophila**,* the polarity is arranged to avoid accumulation of damaged proteins in the longest-lived daughter cell. This pattern of distribution may also be conserved in humans, where we found that aggregated mutant ataxin-3 is absent in the long-lived stem cells of the intestinal crypts but present in a large fraction of the shorter-lived, more differentiated daughter cells. Although the ataxin-3 protein is known to be ubiquitously expressed [41] and was also detected in stem cells in the intestine (Figure 4), we cannot exclude the possibility that the lack of large aggregates in the stem cells is possibly also due to a lower expression of the protein. If the principle of asymmetric distribution of damage indeed holds true for proliferating tissues, it may enable mitotically competent cells to avoid the risk of accumulating toxic protein aggregates during ageing, or in the case of protein misfolding diseases, thus preserving the cells' capacity to produce progeny.

In conclusion, we hypothesise that the principle of asymmetric disposal of damaged cellular proteins during cell division may represent an evolutionarily conserved mechanism to ensure fitness of newly born cells, which is crucial for population expansion, tissue development, and tissue homeostasis.

## Materials and Methods

### Plasmids.

For the transient transfection of mammalian cells, we used the plasmid pEGFP-HDQ74 driving the expression of a fragment of exon-1 of Htt fused to the enhanced green fluorescent protein (EGFP) (kindly provided by David C. Rubinsztein, Cambridge, United Kingdom) and pHDQ119-EYFP (enhanced yellow fluorescent protein), which drive the expression of a fragment of exon-1 of Htt fused to EYFP. These fragments were created by PCR amplification of the Htt-Q128 fragment from isolated genomic DNA of the UAS-Htt-Q128C transgenic fly, kindly provided by J. T. Littleton (Massasachusetts Institute of Technology, United States) [25]. We used the primers HDfor—cgaattcgccaccATGAAGGCCTTCGAGTCCCTCAAGT—and HDrev—GACCGGTACACGGTCTTTCTTGGTAG—that were subsequently cloned in the EcoRI and AgeI sites of pEYFP-N1 vector (Clontech, Palo Alto, California, United States). The plasmid pCDNA-3-H2B-mRFPruby encoding histone H2B that was fused to the monomeric red fluorescent protein (RFP) ruby [42] was created by subcloning the KpnI and StuI fragment from pAc5.1-H2B-mRFPruby [43] into KpnI and EcoRV sites of pCDNA3.1(+) vector (Invitrogen, Carlsbad, California, United States).

### Cell culture and transient transfections.

Hamster O23 lung fibroblast cells and HEK293 cells were cultured in DMEM (Gibco, San Diego, California, United States) supplemented with 10% foetal bovine serum (Sigma, St. Louis, Missouri, United States) and 100 units/ml penicillin and 100 μg/ml streptomycin (Invitrogen). Cultures were maintained at 37 °C and 5% CO_2_ in a humidified incubator. For transient transfections, cells were grown to 50–60% confluence in 35-mm-diameter dishes on coverslips for confocal microscopy analyses and/or round glasses for time-lapse imaging. Cells were transfected with 1 μg of DNA using Lipofectamine (Gibco) according to the manufacturer instructions.

### Microscopy.

Indirect immunofluorescence of γ-tubulin or α-tubulin was performed as described [44]. 16–24 h after transfection to detect centrosomes or microtubules, respectively. Microfilaments were detected likewise by staining of vimentin. Briefly, cells were fixed with methanol:acetone (3:1) for 10 min for γ-tubulin and vimentin detection, or 3.7% formaldehyde for 15 min for α-tubulin and vimentin detection, washed three times with phosphate-buffered saline (PBS), permeabilised with 0.2% Triton-X100, and blocked during 30 min with 0.5% bovine serum albumin (BSA) and 0.1% glycine in PBS. Incubation with rabbit anti–γ-tubulin polyclonal antibody (Sigma T3559), mouse anti–α-tubulin monoclonal antibody (Sigma T5168) at a 1/50 dilution, and/or rat anti–α-tubulin monoclonal antibody (Abcam PLC, Cambridge, United Kingdom) at a 1/500 dilution was performed overnight at 4 °C or for 2 h at room temperature followed by a 1-h incubation with CY3-conjugated anti-rabbit secondary antibody (Amersham Biosciences, Little Chalfont, United Kingdom) at 1:200 dilution, CY5-conjugated anti-mouse secondary antibody (Jackson ImmunoResearch, West Grove, Pennsylvania, United States), or CY3-conjugated anti-rat secondary antibody (Molecular Probes, Eugene, Oregon, United States). For microfilaments staining, 2-h incubations with mouse anti-vimentin (V9) antibody (Santa Cruz Biotechnology, Santa Cruz, California, United States) at a 1/50 dilution was performed followed by 1-h incubation with anti-mouse CY5-conjugated antibody (Jackson) at a 1/200 dilution. To visualise nuclei, cells were stained for 10 min with 0.2 μg/ml DAPI. Coverslips were mounted in antifadent solution (10% Mowiol 40–88 [Sigma], 2.5% 1,4-Diazabicyclo[2.2.2]octane [DABCO, Sigma], and 25% glycerol in 0.1 M Tris-HCl with a pH of 8.5). Images of EGFP, CY3, and DAPI fluorescence were obtained using the Leica confocal laser scanning microscope (Leica TCS SP2, DM RXE, Wetzlar, Germany) with a 63X/1.32 oil lens. The captured images were processed using Leica Confocal Software and Adobe Photoshop.

### Time-lapse imaging.

Transfected cells were grown on round coverslips for 16 h. The coverslip was transferred to a chamber that was especially designed for life imaging on our Leica TCS microscope, which was filled with 1 ml medium. The chamber was then placed in a temperature- and gas-controlled incubator (37 °C and 5% CO_2_) on the microscope stage (temperature kept at 37 °C) of an inverted Leica confocal laser scanning microscope (Leica TCS SP2, DM IRBE). To prevent cooling, the lens was also maintained at 37 °C. Images were made in 14 optical planes (approximately 1 μm thick) every 10 min for a period of 16–30 h. O23 cells expressing the EGFP fusion protein were excited with a 488-nm Argon laser, and emission was measured with a band pass 500–550-nm filter. HEK293 cells co-expressing the EYFP and mRFPruby fusion proteins were excited with a 488-nm Argon laser and a 543-nm helium-neon laser, and emission was measured with a BP 500–550-nm filter in combination with a TD 488/543/633 splitter. After time-lapse imaging, slices of the vertical sections were converted to projected images using the Leica Confocal Sofware.

### Drosophila melanogaster stocks and genetics.

*Drosophila* stocks were maintained at room temperature according to standard protocols. The UAS-Htt-Q128C–expressing stock was kindly provided by JT Littleton (Massasachusettes Intitutue of Technology, United States) [25], the UAS-Pon-GFP-5 expressing stock was a gift from YN Jan (Howard Hughes Medical Institute, San Francisco, California, United States) [26], and the neur-GAL4-A101 was obtained from the Bloomington Stock Center (http://flystocks.bio.indiana.edu/; stock number 6393). UAS-Htt-Q128C and UAS-Pon-GFP-5, were recombined onto the same second chromosome by meiotic recombination. Expression of Htt-Q128C and Pon-GFP-5 was confirmed by Western blotting and single fly PCR analysis.

### *Drosophila* embryonic neuroblasts isolation, culture, and immunofluorescence.

Antibody staining of embryos was performed as described previously [45]. Neuroblasts isolation was performed as described [32]. Briefly, 1–5-h-old embryos were dechorionated, rinsed for 5 min in 95% ethanol, and equilibrated in Schneider's insect medium plus 2% fetal calf serum (Gibco). Embryos were homogenised by six to eight strokes of a glass Tenbroek, and the cell suspension was filtered in a 5-ml polystyrene round-bottom tube with cell-strainer (35 μm) cap (Becton, Dickinson, and Co., Franklin Lakes, New Jersey, United States). Cells were pelleted at 1000 revolutions per minutes and washed twice with fresh medium. Dissociated cell suspension (0.5 ml) was plated on coverslips, and cells were allowed to adhere to the coverslips for 30 min prior to the addition of 1.5 ml of medium. Cultures were grown for 2–3 h at 25 °C. Cells were fixed for 10 min in 3.7% formaldehyde in PBS, washed once with PBS, permeabilised with 0.2% Triton-X100, and blocked 30 min with 0.5% BSA and 0.1% glycine in PBS. Incubation with mouse anti-huntingtin monoclonal antibody (Chemicon, Temecula, California, United States), at a 1/500 dilution alone or in combination with rat anti–α-tubulin antibody (Abcam) at a 1/500 dilution was performed overnight at 4 °C, followed by 1 h incubation with CY5-conjugated goat anti-mouse antibody (Jackson) at 1/200 dilution, and CY3-conjugated anti-rat antibody (Molecular Probes) at 1/200 dilution. Nuclear staining, mounting, and visualisation were performed in the same way as described for mammalian cells.

### Immunohistochemistry and electron microscopy.

Human intestine tissue that was obtained from deceased SCA3 patients 24 h after death was fixed in 4% para-formaldehyde and cryosectioned in 50-μm sections. Immunohistochemistry of ataxin-3 was performed with a polyclonal antibody against the full-length protein (a generous gift from H. Paulson, Iowa City, United States) diluted 1:5000 and with a polyglutamine-expansion specific mouse 1C2 (Chemicon; MAB 1574) diluted 1:3000. DAB label was generated by GAR/RAM biotin (1:400) and VectaStain ABC (1:400). Negative controls for immunohistochemistry were treated without primary antibodies, and positive labelling for ataxin-3 aggregates was confirmed in pontine neurons. By gold-substituted silver peroxidase, DAB was converted into gold label suitable for electron microscopy. Flat embedding was performed in Epon according to routine procedures. Light microscopy of Epon sections (1 μm) stained for Toluidin Blue localised the crypts that were subsequently used for electron microscope screening of ultrathin sections in a transmission electron microscope CM100 (Philips, Eindhoven, The Netherlands) at 80 kV. The same was done for control tissue obtained from an autopsy of a healthy individual. Detection of stem cells in the intestinal crypts was performed in 4-μm sections of paraffin embedded tissue or 1 μm cryosections using rabbit polyclonal anti-Musashi-1 antibody (1:200; Chemicon; AB5977). Visualisation was done with DAB generated by avidin-biotin-horseradish peroxidase complex (Vector Elite Avidin-Biotin Complex kit), anti-rabbit biotin conjugated antibody (1:300; DAKO, E0431). 1 mM EDTA antigen retrieval was performed in paraffin sections prior to antibodies labelling.

## Supporting Information

Figure S1 Polyglutamine-Expanded Proteins Form Aggresomes in Human HEK293 Cells (A) Aggresome-like inclusions are either close to (upper panel) or colocalise (lower panel) with the centrosomes (decorated with γ-tubulin antibodies) in interphase HEK293 cells.
(B) Vimentin microfilaments are redistributed in a cage-like manner around the inclusion, consistent with aggresome morphology. The cell in the lower panel corresponds to the confocal planes shown in Figure 1E. DNA is stained with DAPI (blue). Bars represent 10 μm.
(5.0 MB TIF)

Figure S2 Secondary Inclusions Do Not Associate with Centrosomes, and Spindle Midbodies Organise Normally in Cells with Aggresomes (A) Secondary inclusions do not associate with centrosomes. Expression of an EGFP-tagged polyglutamine-expanded huntingtin fragment (EGFP-HDQ74) (green) not only induces inclusion formation at centrosomes (aggresomes: Figure 1), but in some cases, also leads to the formation of secondary large and/or small inclusions throughout the cell that do not localise with the centrosomes. Immunolabelling of γ-tubulin (red) was used to visualise the centrosomes and nuclei are stained with DAPI (blue). Bar, 20 μm.
(B) Spindle midbodies organise normally in cells with aggresomes. Spindle midbodies stained with anti-γ-tubulin antibodies (red) have normal morphology in cells with a diffuse distribution of (EGFP-HDQ74) (green) (upper panel) and in aggresome containing cells (middle panel), whereas in cells with scattered multiple inclusions, the spindle midbody is thickened and disorganised (lower panel). DNA is stained with DAPI (blue). Bars, 10 μm.
(3.3 MB TIF)

Video S1 Aggresome Formation Does Not Impair Cell Division in O23 Cells and Leads to Asymmetric Inheritance of Aggregated Proteins Video of an O23 cell expressing an EGFP-tagged polyglutamine-expanded huntingtin fragment (EGFP-HDQ74) that forms an aggresome, enters mitosis, and completes cytokinesis within 40 min. Images were taken at 10-min intervals, and the frame rate is two images per second. The video corresponds to Figure 3A.
(9.0 MB AVI)

Video S2 Aggresome Formation Does Not Impair Cell Division in O23 Cells and Leads to Asymmetric Inheritance of Aggregated Proteins Video of a O23 cell expressing an EGFP-tagged polyglutamine-expanded *huntingtin* fragment (EGFP-HDQ74) that forms an aggresome, enters mitosis, and completes cytokinesis within 40 min. Images were taken at 10-min intervals, and the frame rate is two images per second.
(6.3 MB AVI)

Video S3 Aggresome Formation Does Not Impair Cell Division in HEK293 Cells and Leads to Asymmetric Inheritance of Aggregated Proteins Video of a HEK293 cell expressing an EYFP-tagged polyglutamine-expanded huntingtin fragment (HDQ119-EYFP) (green) and H2B-mRFP ruby for visualisation of the DNA that have an aggresome, enter mitosis, and complete cytokinesis within 80 min. Images were taken at 10-min intervals, and the frame rate is two images per second. The video corresponds to Figure 3B.
(2.7 MB AVI)

Video S4 Aggresome Formation Does Not Impair Cell Division in HEK293 Cells and Leads to Asymmetric Inheritance of Aggregated Proteins Video of a HEK293 cell expressing an EYFP-tagged polyglutamine-expanded huntingtin fragment (HDQ119-EYFP) (green) and H2B-mRFP ruby for visualisation of the DNA that have an aggresome, enter mitosis, and complete cytokinesis within 80 min. Images were taken at 10-min intervals, and the frame rate is two images per second.
(977 KB AVI)

Video S5 Mitosis is Extremely Delayed in Cells with Multiple Inclusions Video of an O23 cell expressing an EGFP-tagged polyglutamine-expanded huntingtin fragment (EGFP-HDQ74) with an aggresome that subsequently forms other inclusions, especially in the nucleus. The cell enters mitosis and completes cytokinesis, but compared to cells with a single aggresome (Figure 3 and Videos S1 and S2), cytokinesis is severely delayed. Images were taken at 10-min intervals, and the frame rate is two images per second. The video corresponds to Figure 3D.
(11 MB AVI)

Video S6 Mitosis in a Diffuse EGFP-HDQ74–Expressing O23 Cell Video of a cell expressing the EGFP-tagged polyglutamine-expanded huntingtin fragment (EGFP-HDQ74) that shows a diffuse distribution (i.e., still without any detectable aggregates). The cell enters mitosis and completes cytokinesis in about 30 min. Images were taken at 10-min intervals, and the frame rate is two images per second.
(4.4 MB AVI)

Video S7 Mitosis in a Diffuse HDQ119-Expressing HEK293 cell Video of a cell expressing the EYFP-tagged polyglutamine-expanded huntingtin fragment (EGFP-HDQ119) that shows a diffuse distribution (i.e., still without any detectable aggregates). The cell enters mitosis and completes cytokinesis in about 70 min. Images were taken at 10-min intervals, and the frame rate is two images per second.
(1.5 MB AVI)

### Accession Numbers

The Online Mendelian Inheritance in Man (http://www.ncbi.nlm.nih.gov/entrez/query.fcgi?db=OMIM) accession number for SCA3 is 109150.

## Abbreviations

EGFP: enhanced green fluorescent protein
EYFP: enhanced yellow fluorescent protein
GFP: green fluorescent protein
GMC: ganglion mother cell
HEK 293: human embryonic kidney 293
Htt: huntingtin
MTOC: microtubule organizing center
Pon: Partner of Numb
SCA3: spinocerebellar ataxia type 3
