# Supplementary figures and images for: Polarised Asymmetric Inheritance of Accumulated Protein Damage in Higher Eukaryotes
Source: PLoS Biol. 2006 Dec 5;4(12):e417. doi: 10.1371/journal.pbio.0040417 (PMC1750924; doi:10.1371/journal.pbio.0040417)

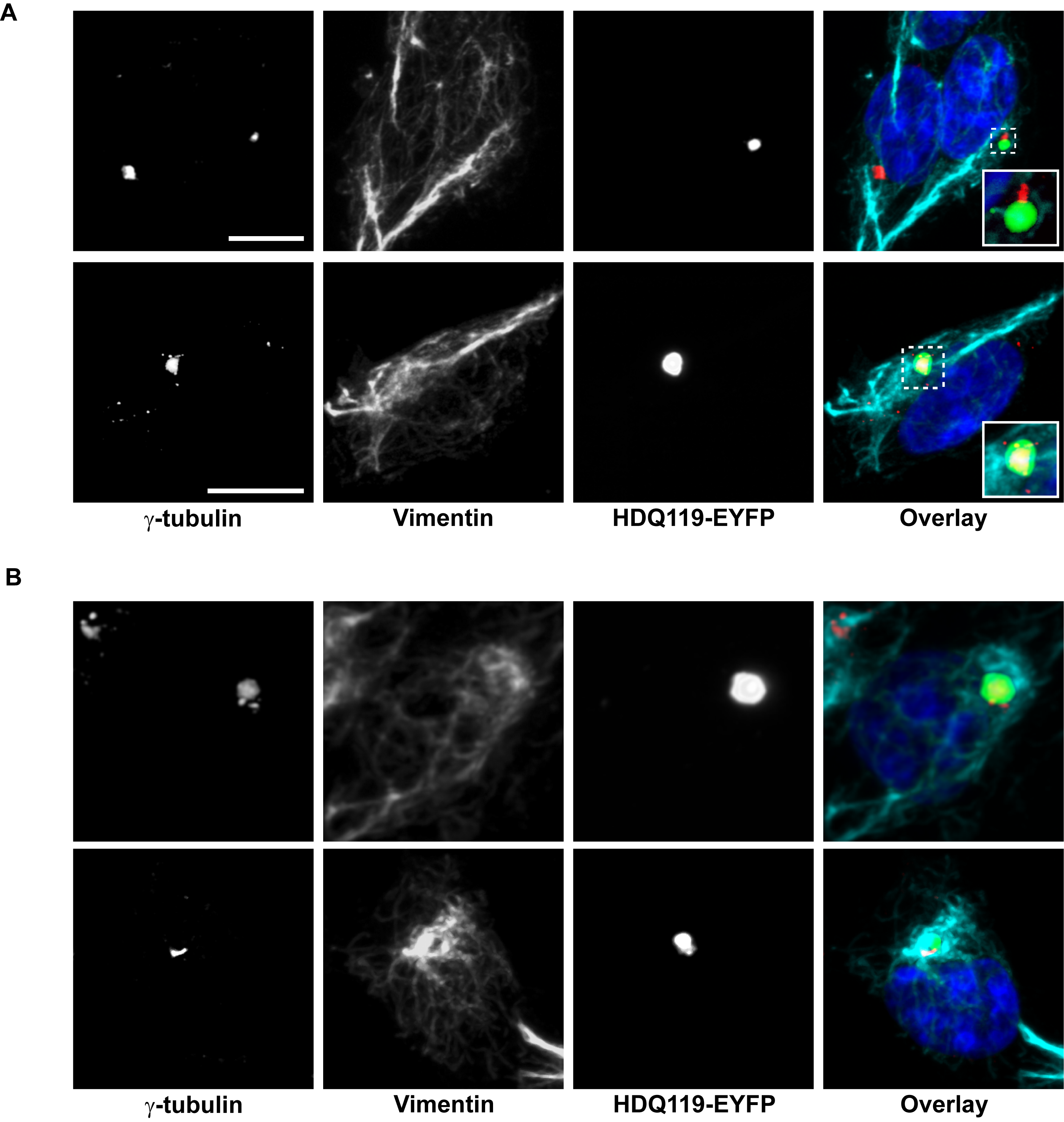

Supplement: Figure S1 — (A) Aggresome-like inclusions are either close to (upper panel) or colocalise (lower panel) with the centrosomes (decorated with γ-tubulin antibodies) in interphase HEK293 cells. (B) Vimentin microfilaments are redistributed in a cage-like manner around the inclusion, consistent with aggresome morphology. The cell in the lower panel corresponds to the confocal planes shown in Figure 1E. DNA is stained with DAPI (blue). Bars represent 10 μm. (5.0 MB TIF) [file pbio.0040417.sg001.tif]

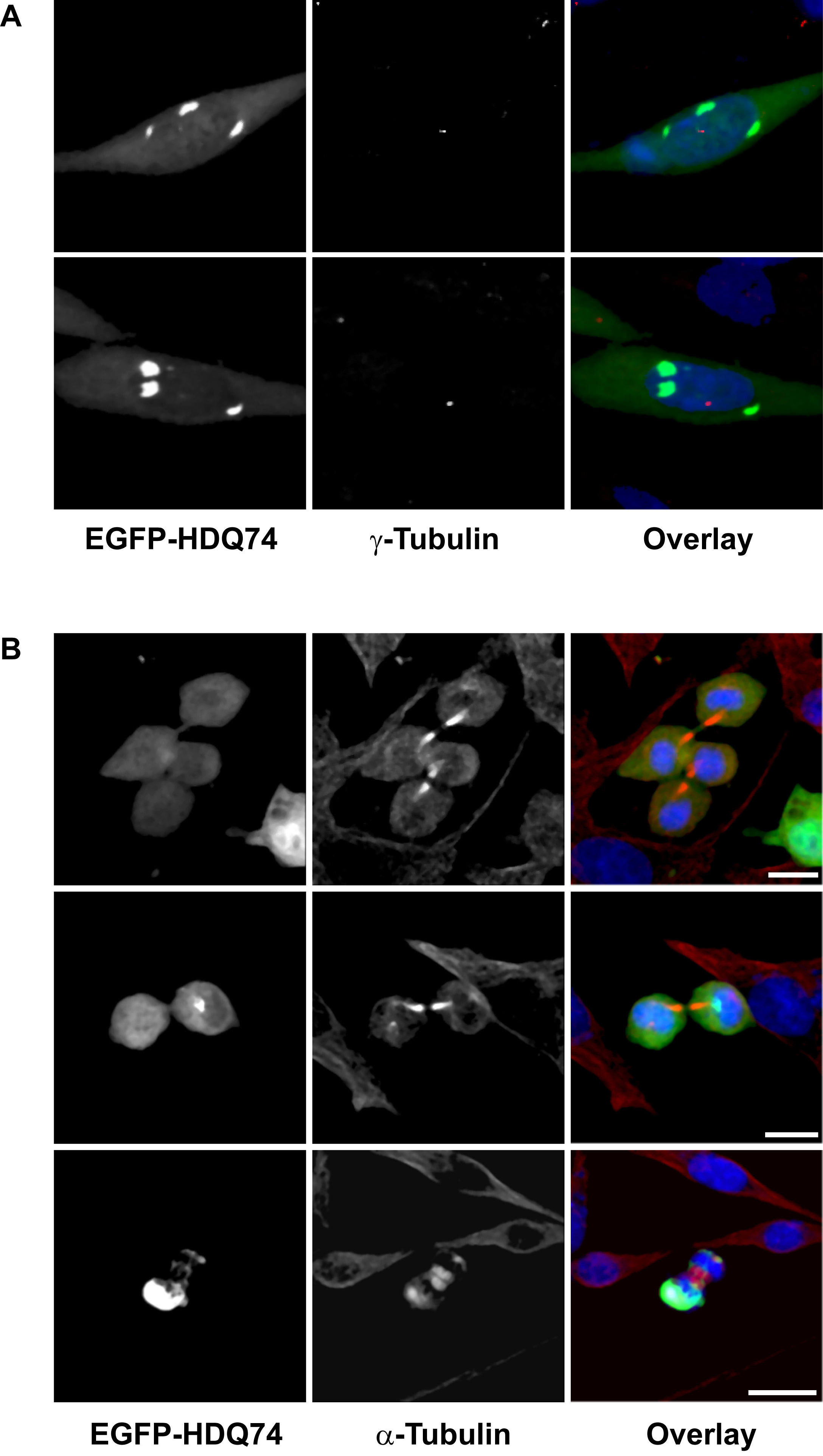

Supplement: Figure S2 — (A) Secondary inclusions do not associate with centrosomes. Expression of an EGFP-tagged polyglutamine-expanded huntingtin fragment (EGFP-HDQ74) (green) not only induces inclusion formation at centrosomes (aggresomes: Figure 1), but in some cases, also leads to the formation of secondary large and/or small inclusions throughout the cell that do not localise with the centrosomes. Immunolabelling of γ-tubulin (red) was used to visualise the centrosomes and nuclei are stained with DAPI (blue). Bar, 20 μm. (B) Spindle midbodies organise normally in cells with aggresomes. Spindle midbodies stained with anti-γ-tubulin antibodies (red) have normal morphology in cells with a diffuse distribution of (EGFP-HDQ74) (green) (upper panel) and in aggresome containing cells (middle panel), whereas in cells with scattered multiple inclusions, the spindle midbody is thickened and disorganised (lower panel). DNA is stained with DAPI (blue). Bars, 10 μm. (3.3 MB TIF) [file pbio.0040417.sg002.tif]
